# Jejunal lipoma presenting as small bowel intussusception in an adult: diagnostic and surgical challenges—a case report

**DOI:** 10.1093/jscr/rjaf551

**Published:** 2025-09-24

**Authors:** Midani Touati, Faiez Boughanmi, Hiba Ben Hassine, Hanene Zenati, Leith Limayem, Faouzi Noomen

**Affiliations:** Department of Visceral Surgery, Fattouma Bourguiba Hospital, Monastir, Tunisia; Department of Visceral Surgery, Fattouma Bourguiba Hospital, Monastir, Tunisia; Department of Visceral Surgery, Fattouma Bourguiba Hospital, Monastir, Tunisia; Department of Visceral Surgery, Fattouma Bourguiba Hospital, Monastir, Tunisia; Department of Visceral Surgery, Fattouma Bourguiba Hospital, Monastir, Tunisia; Department of Visceral Surgery, Fattouma Bourguiba Hospital, Monastir, Tunisia

**Keywords:** adult intussusception, jejunal lipoma, benign gastrointestinal tumors, case report

## Abstract

Intussusception is a rare cause of adult small bowel obstruction, often associated with malignant lesions but occasionally caused by benign etiologies like lipomas. The clinical presentation is diverse, with symptoms ranging from abdominal pain to changes in bowel habits. Computed tomography plays a pivotal role in diagnosis. Surgical intervention remains the cornerstone of treatment, involving resection of the affected bowel segment and removal of underlying lesions. This case report presents a unique occurrence of intussusception caused by jejunal lipoma in a 27-year-old male, highlighting its clinical features, diagnostic approach, and the crucial role of timely surgical intervention for optimal patient outcomes.

## Introduction

Intussusception in adults, especially caused by a jejunal lipoma, is an uncommon condition presenting diagnostic challenges due to its infrequent occurrence and diverse clinical manifestations [1]. Unlike in children, where intussusception is often idiopathic, adult intussusception typically has an identifiable cause, “lead points,” with benign and malignant neoplasms [2, 3]. While neoplasms are common lead points, jejunal lipomas causing intussusception are exceedingly rare. The diagnosis of adult intussusception can be challenging due to its subtle clinical presentation. High clinical suspicion is necessary, and imaging studies such as computed tomography (CT) scans aid in the diagnosis [3]. The treatment of adult intussusception typically involves surgical intervention. We report a rare case of adult intussusception caused by a jejunal lipoma.

## Case presentation

A 27-year-old male presented to the emergency department with complaints of nausea, vomiting, and intermittent upper abdominal pain. The patient reported experiencing similar episodes over the past 6 weeks, which resolved spontaneously. No history of peptic ulcer disease, bowel habit changes, melena, or weight loss was reported. On examination, he was afebrile, hemodynamically stable, with a distended abdomen, no rigidity, rebound tenderness, or palpable masses. Digital rectal examination revealed an empty rectal vault with no masses or evidence of bleeding.

**Figure 1:**
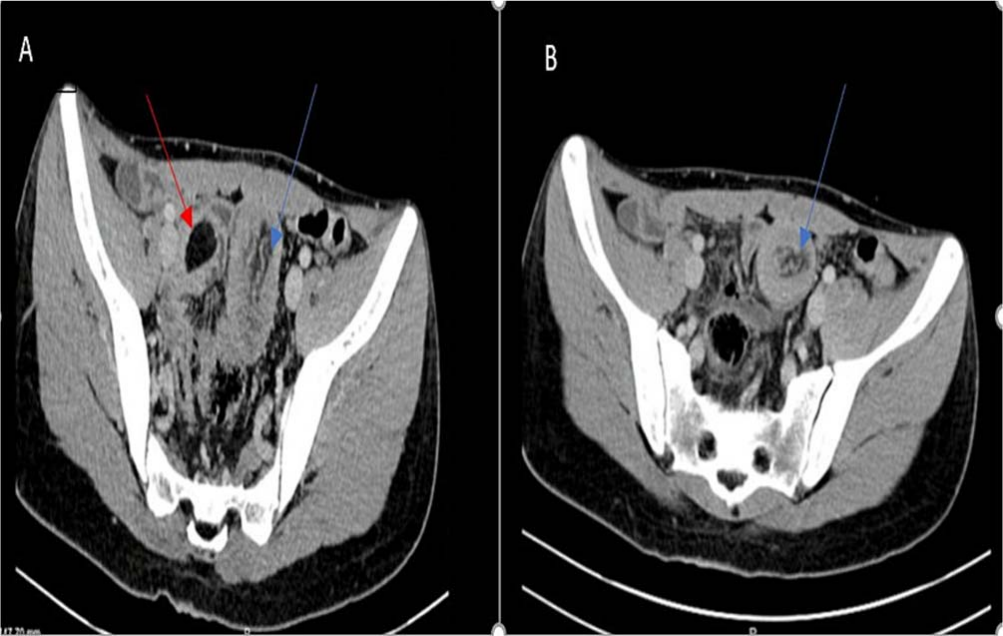
Axial CT scan showing an oval fat density mass representing a jejunal lipoma (A) and a telescoping intestine measuring 20 × 4 cm (B).

**Figure 2:**
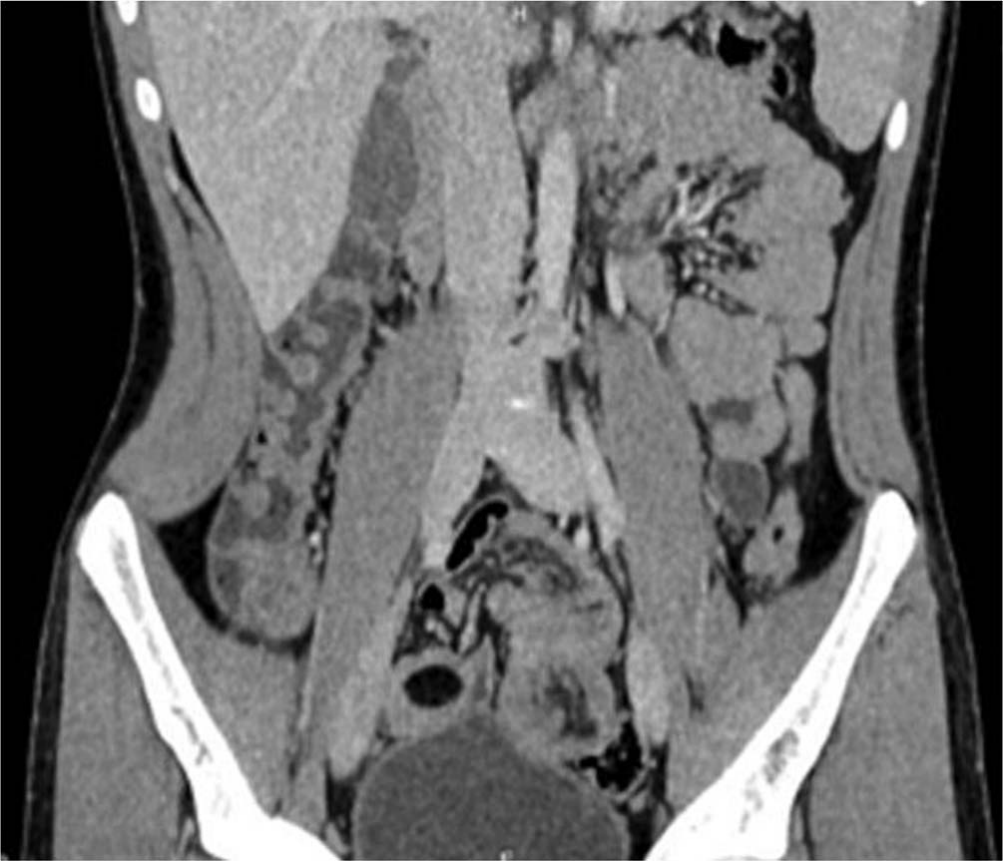
Coronal CT scan showing “sausage-shaped” mass.

Laboratory findings were within normal ranges. Abdominal CT revealed a telescoping intestine measuring 20 × 4 cm, with an intraluminal lesion displaying fat-density at 15 × 25 mm (Fig. 1), surrounded by the thick-walled intussuscipiens (Fig. 2). No significant proximal bowel dilation or additional lesions were observed.

These findings led to a diagnosis of intussusception induced by a jejunal lipoma.

**Figure 3:**
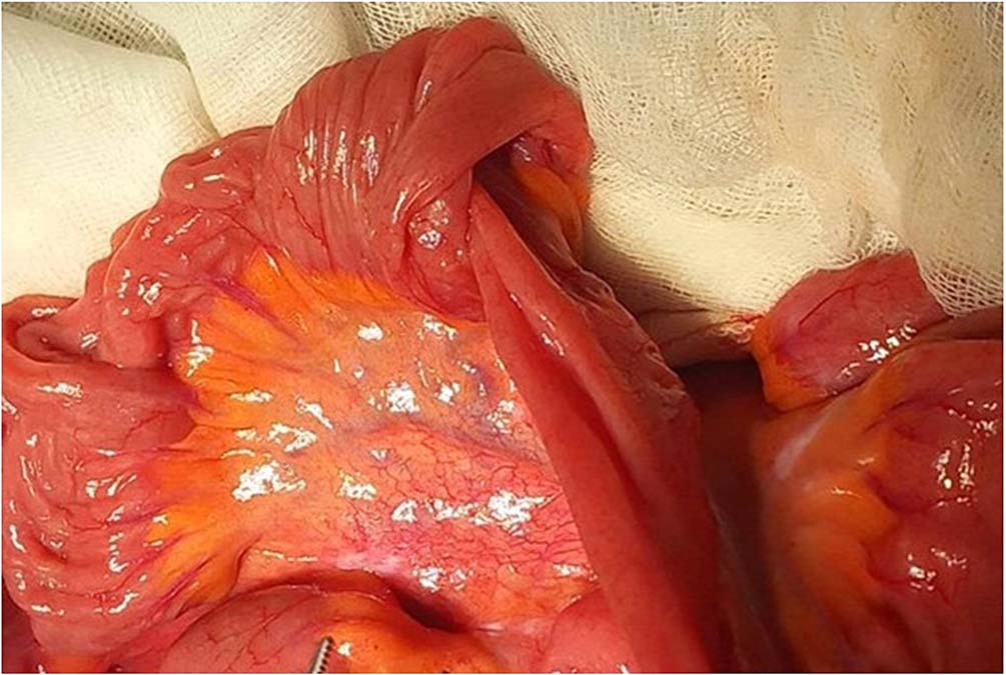
Preoperative view of compounded ileo-ileal intussusception.

**Figure 4:**
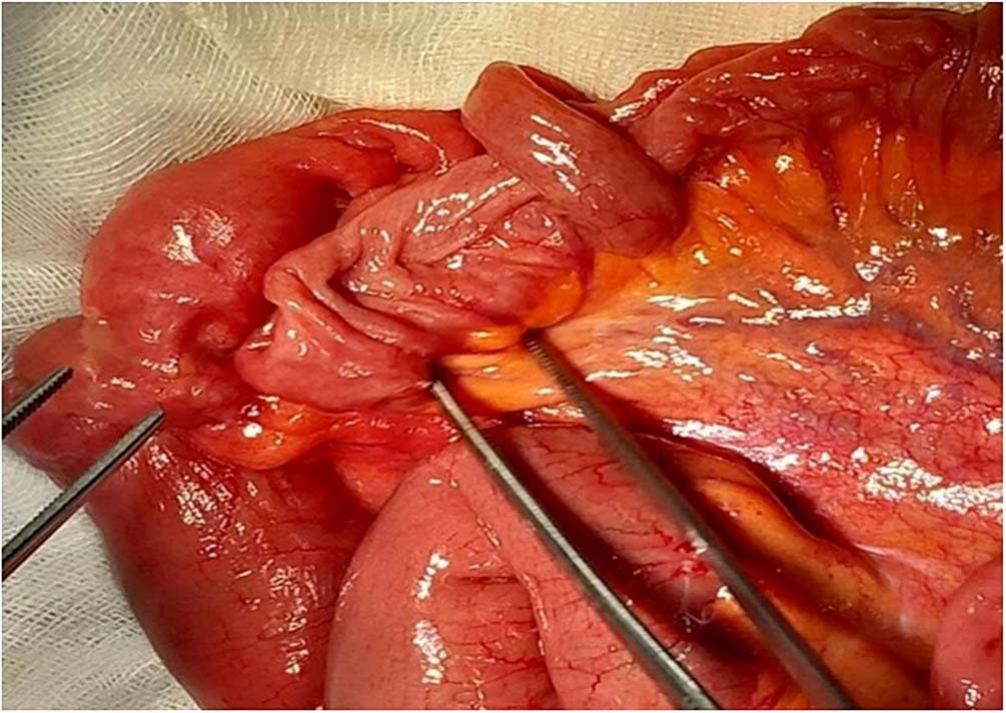
Preoperative view of manual reduction exposed a compound ileo-ileal intussusception without vascular compromise.

**Figure 5:**
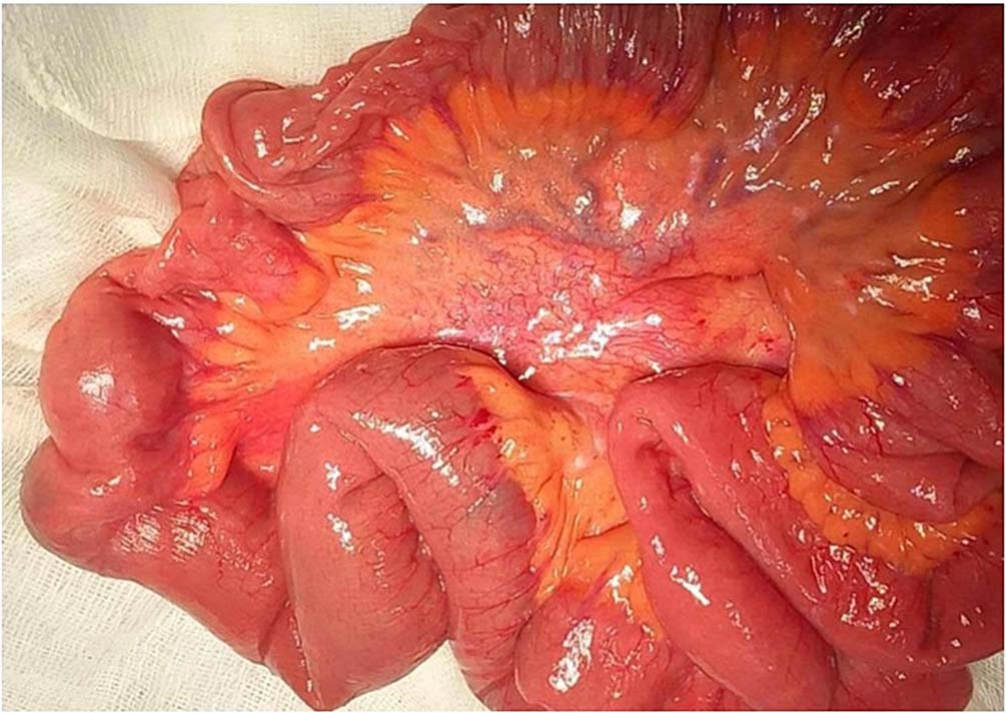
Preoperative view of submucosal lipoma.

Emergency laparotomy revealed a jejuno-jejunal intussusception 60 cm distal to the ligament of Treitz (Fig. 3). Manual reduction exposed a compound ileo-ileal intussusception without vascular compromise (Fig. 4). The intramural lipoma was subsequently identified (Fig. 5). Resection and end-to-end anastomosis of the involved ileal segment, with a 5-cm safety margin on each side, were performed.

Histopathological examination confirmed a submucosal intestinal lipoma with no dysplasia or malignancy. The postoperative course was uneventful, and the patient was discharged after 5 days.

## Discussion

Intussusception is defined as the invagination of a segment of bowel with its mesenteric fold (intussusceptum) into an adjacent intestinal segment (intussuscipiens) [4]. It is a common pediatric condition and occurs relatively rarely in adults. Intussusception in adults is seen in only 1%–5% of small bowel obstructions [4, 5]. While pediatric cases are often idiopathic, 90% of cases of adult intussusception frequently result from organic lead points [1]. The lead point of small bowel intussusception is malignant in 64% of cases [2, 3]. Benign causes, including various polyps (Peutz-Jeghers, hamartomatous, fibrous, inflammatory), diverticula (Meckel’s), and small bowel lipoma, are more uncommon. The etiology involving a lipoma, as observed in our case, is exceptional, representing only 1%–2% of all gastrointestinal tumors [6, 7]. Lipomas are benign tumors of mesenchymal origin found anywhere along the gastrointestinal tract, with the most common location being the terminal ileum. Lipomas of the bowel are usually submucosal, intramuscular, and subserosal are less common, with no malignant potential. The peak age of incidence is in the 6th–7th decades of life, and it appears that females are more prone to lipomas. Preoperative diagnosis is usually missed or delayed because of nonspecific and often sub-acute symptoms. The onset of clinical manifestations is correlated with the size of lipoma (from 4 cm onwards), which is responsible for acute pain; occult bleeding from mucosal ulceration, and intestinal intussusception [7]. Adult intussusception typically presents with gradual onset symptoms, contrasting with the acute manifestation in children. Less than 20% of cases manifest with acute symptoms leading to complete bowel obstruction. Additionally, a palpable abdominal mass is only found in a relatively small percentage, ranging from 7% to 42% of cases [8]. Specific symptoms like colicky abdominal pain, nausea, vomiting, and bleeding are nonspecific, leading to delayed diagnosis and treatment, often identified during surgery. Imaging, particularly abdominal CT, is considered the most sensitive imaging modality in the diagnosis of intussusception and distinguishes the presence or absence of a lead point. It has a sensitivity of 71%–87% and a specificity of up to 100% [9]. CT scans show a typical appearance of bowel-within-bowel configuration with or without contained fat and mesenteric vessels. This appears as a “target” or a “doughnut” mass in images vertical to the longitudinal axis of the lesion or as a “sausage-shaped” mass parallel to its longitudinal axis. An advantage of CT is that any associated intussusception is also revealed, and vascularity of the bowel can be assessed [9]. Due to the fact that adults’ intussusception is often associated with malignant organic lesions, emergency surgery is the recommended management. The choice of surgical intervention depends on tumor presence and intestinal necrosis. The treatment plan for adult intussusception consists of segmental resection and primary restoration of the continuity of the gastrointestinal tract [10]. Oncological principles should be maintained during resection unless pre-operative imaging shows a benign etiology. However, the extent of resection and whether or not the intussusception should be initially reduced remain controversial. Manual reduction by squeezing the intussusception is suitable for ileo-ileal intussusception without necrosis or tumor, whereas intestinal resection is necessary otherwise. However, when feasible, prior reduction can help better define the resection boundaries and occasionally reduce its extent, especially in cases of benign tumors. Objections to reductions are theoretically based on: the possibility of intraluminal seeding and venous dissemination of malignant cells; possible perforation during manipulation; and the increased risk of anastomotic complications in the presence of oedematous and inflamed bowel [11]. An anatomopathological study is necessary for diagnostic confirmation. Treatment is usually definitive; no malignant transformation of lipomas has been reported in the literature, and after resection, no recurrence is expected.

Adult intussusception caused by a jejunal lipoma is a rare condition, challenging to diagnose due to its intermittent and nonspecific symptoms. It is important to consider this possibility in patients presenting with chronic or intermittent symptoms suggestive of intestinal obstruction. Surgical resection stands as the primary treatment modality and generally results in a favorable prognosis for affected individuals. Reduction before resection can be attempted in small bowel intussusceptions, provided that the segment involved is viable, or a malignancy is not suspected. Surgeons should be familiar with the various treatment options because the real cause of the intussusception is often only accurately diagnosed at laparotomy.

## Conclusion

Adult intussusception caused by a jejunal lipoma is a rare but important differential diagnosis in patients with intermittent abdominal symptoms. Despite its nonspecific clinical presentation, timely diagnosis using CT imaging and surgical management through segmental resection offers excellent outcomes. Awareness of benign causes such as lipomas can prevent delays in treatment and unnecessary extensive resections.
